# Pathogenic and Regulatory Roles of Fibrinolytic Factors in Autoimmune Diseases

**DOI:** 10.3390/cimb47100790

**Published:** 2025-09-23

**Authors:** Yosuke Kanno

**Affiliations:** Department of Molecular Pathology, Faculty of Pharmaceutical Science, Doshisha Women’s College of Liberal Arts, 97-1 Kodo Kyotanabe, Kyoto 610-0395, Japan; ykanno@dwc.doshisha.ac.jp

**Keywords:** fibrinolytic factor, autoimmune diseases, plasmin, plasminogen, plasminogen activator, alpha2-antiplasmin, PAI-1

## Abstract

Autoimmune diseases arise from complex interactions of genetic, environmental, and hormonal factors, yet their precise causes remain elusive. Beyond its canonical role in fibrin degradation, the fibrinolytic system is increasingly recognized as both a pathogenic driver and a regulatory modulator in autoimmunity. Key factors—plasminogen (Plg), plasmin, α2-antiplasmin (α2AP), tissue-type plasminogen activator (tPA), urokinase-type plasminogen activator (uPA), its receptor (uPAR), and plasminogen activator inhibitor-1 (PAI-1)—not only reflect secondary responses to vascular and immune dysregulation but also actively shape innate and adaptive immunity. They influence macrophage activation, dendritic cell maturation, T cell responses, and cytokine production, thereby bridging coagulation, inflammation, and tissue repair. This review integrates current evidence on the dual pathogenic and regulatory roles of fibrinolytic factors, organizing autoimmune diseases into systemic, organ-specific, and secondary syndromes. We further discuss how the imbalance of fibrinolysis can either promote inflammatory persistence or, conversely, facilitate resolution through fibrin clearance and immune homeostasis. By highlighting this bidirectional influence, the review aims to refine our understanding of fibrinolytic components as both contributors to and regulators of autoimmune disease pathogenesis.

## 1. Introduction

Autoimmune diseases are a group of disorders in which the immune system mistakenly attacks the body’s own tissues and organs. The onset and progression of these conditions—including rheumatoid arthritis (RA), systemic lupus erythematosus (SLE), systemic sclerosis (SSc), and type 1 diabetes (T1D)—are thought to involve a complex interplay of genetic, environmental, and hormonal factors.

Disease progression is driven by autoreactive lymphocytes (B cells and T cells) that cause tissue and organ damage. These lymphocytes undergo polyclonal expansion, meaning they express diverse antigen receptors capable of recognizing a wide array of self-antigens, thereby sustaining chronic inflammation and promoting pathological tissue injury. In addition to adaptive immune responses, dysregulation of the innate immune system—including macrophages, dendritic cells, and neutrophils—also plays a pivotal role in disease pathogenesis. Nevertheless, the precise etiology of autoimmune diseases remains incompletely understood.

**Figure 1:**
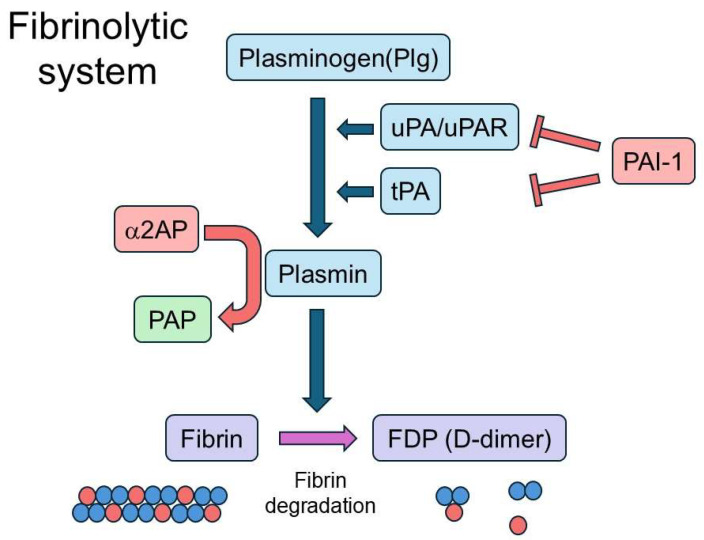
Fibrinolytic system. Plg is converted to plasmin by uPA bound to its receptor (uPAR) or by tPA. Plasmin degrades fibrin into fibrin degradation products (FDPs, including D-dimer). Plasmin activity is inhibited by α2AP, while plasminogen activators (uPA/uPAR, tPA) are inhibited by PAI-1. PAP and D-dimer serve as a biomarker of fibrinolytic activity.

Recent studies have revealed that the fibrinolytic system, traditionally recognized for its role in fibrin degradation and vascular homeostasis, also plays a critical role in modulating immune responses. Importantly, fibrinolytic components can function not only as secondary byproducts of immune activation but also as pathogenic drivers or regulatory “gatekeepers” that shape disease outcomes. The fibrinolytic system includes plasmin and its precursor plasminogen (Plg), as well as tissue-type plasminogen activator (tPA), urokinase-type plasminogen activator (uPA), and its receptor (uPAR), which collectively promote plasmin generation. It also comprises α2-antiplasmin (α2AP), a primary inhibitor of plasmin, and plasminogen activator inhibitor-1 (PAI-1), which inhibits both tPA and uPA (Figure 1).

These factors are involved in regulating both innate and adaptive immunity. Despite growing evidence linking fibrinolytic factors to immune dysregulation and fibrosis, the specific mechanisms by which these components influence autoimmune disease progression remain poorly understood. Moreover, their impact appears context-dependent, with fibrinolytic imbalance either amplifying inflammation or, conversely, facilitating resolution through timely fibrin clearance. This underscores the need for a comprehensive and disease-specific analysis.

To address the diverse roles of fibrinolytic dysregulation in immune-mediated diseases, this narrative review synthesizes current knowledge on the involvement of fibrinolytic factors in autoimmune pathophysiology. Diseases were selected based on the availability of mechanistic and clinical evidence retrieved through PubMed searches.

To guide the literature selection, we used the following inclusion criteria: (1) articles published in peer-reviewed journals, (2) studies focusing on fibrinolytic components (e.g., plasmin, PAI-1, uPA/uPAR, α2-antiplasmin) in the context of autoimmune diseases, and (3) both original research and review articles that provide mechanistic insights or clinical relevance. There were no restrictions on study design, patient population, or publication date. Non-English articles and those lacking mechanistic or clinical data were excluded.

We categorized autoimmune conditions into three groups: systemic autoimmune diseases, organ-specific autoimmune diseases, and secondary syndromes associated with autoimmunity. These categories were chosen to reflect the broad spectrum of fibrinolytic involvement across immune contexts. While not exhaustive, the diseases included represent well-characterized models with established links to the fibrinolytic system.

We begin by providing an overview of the fibrinolytic system, its regulatory mechanisms, and its effects on key immune cell populations. This is followed by a disease-oriented discussion focusing on representative autoimmune diseases from each category. Finally, we examine the therapeutic implications and outline future directions for targeting fibrinolytic pathways in autoimmunity. This review includes 174 original and review articles published between 1991 and 2025, retrieved primarily through PubMed using combinations of search terms such as “fibrinolysis,” “Plg,” “PAI-1,” “uPA/uPAR,” and the names of specific autoimmune diseases. We prioritized peer-reviewed studies that provided mechanistic or translational insight into fibrinolysis and autoimmunity. Case reports, non-English publications, and studies lacking clear mechanistic relevance were excluded. While our approach ensured broad coverage across systemic and organ-specific autoimmune diseases, this is not a systematic review, and the possibility of selection bias remains. The strength of our approach lies in its comprehensive scope and integration of fibrinolysis with immune regulation; however, uneven distribution of available studies across diseases and incomplete mechanistic data represent limitations.

## 2. Fibrinolytic Factors

The fibrinolytic system is a physiological process that maintains circulatory balance by degrading fibrin clots, thereby preventing thrombosis and preserving vascular homeostasis. Plasmin, the central enzyme in this system, is generated from its inactive precursor Plg through cleavage by tPA or uPA and its receptor uPAR. This reaction leads to the production of fibrin degradation products (FDPs), including D-dimer (Figure 1). Beyond clot dissolution, plasmin mediates a range of biological processes, such as the activation of transforming growth factor-β (TGF-β) and vascular endothelial growth factor (VEGF), extracellular matrix (ECM) remodeling, activation of matrix metalloproteinases (MMPs), and modulation of immune responses through cytokine production, protease-activated receptor (PAR) signaling, and regulation of apoptosis [1,2,3,4,5,6,7,8]. tPA, which is primarily secreted by endothelial cells, exhibits fibrin-dependent activity that is essential for clot resolution. It also contributes to angiogenesis and ECM remodeling through interactions with proteins such as low-density lipoprotein receptor-related protein 1 (LRP1) and N-methyl-D-aspartate receptor (NMDAR) [1,9,10]. uPA is expressed in various cell types, including immune and stromal cells. Upon binding to its receptor uPAR, uPA generally promotes cellular processes such as proliferation, migration, inflammation, and angiogenesis in most contexts [1,11,12,13,14]. uPAR further interacts with integrins, epidermal growth factor receptor (EGFR), and vascular endothelial growth factor receptor 2 (VEGFR2), thereby facilitating immune cell adhesion and modulating signal transduction pathways [15,16]. Additionally, uPAR is cleaved by several enzymes, including plasmin, uPA, and MMPs, resulting in the generation of soluble uPAR (suPAR) [17]. suPAR can initiate intracellular signaling and influence various cellular functions, including proliferation, migration, and adhesion. It is also recognized as a biomarker for severe immune and inflammatory diseases [17,18].

To maintain homeostasis, α2AP serves as the primary inhibitor of plasmin by rapidly forming an irreversible complex known as plasmin–α2AP complex (PAP). In addition to limiting fibrinolysis, α2AP is involved in immune modulation, vascular remodeling, and fibrotic processes through various mechanisms, including cytokine production, cell proliferation, and differentiation [5,19,20,21,22,23]. PAI-1, another key inhibitor, suppresses both tPA and uPA activity, thereby stabilizing clots. Moreover, PAI-1 influences cell adhesion and migration by interacting with proteins such as vitronectin and LRP1, promotes wound healing, and modulates macrophage and neutrophil responses during inflammation and tissue repair [5,12,24,25,26,27,28,29,30].

## 3. Immune Activation and Inflammation Mediated by the Fibrinolytic System

Recent evidence indicates that fibrinolytic factors play a critical role in the regulation of both innate and adaptive immunity. Components such as plasmin, uPA, uPAR, α2AP, and PAI-1 modulate immune cell activity, cytokine expression, ECM remodeling, and inflammatory responses in various contexts.

### 3.1. T Cells

Plasmin contributes to T cell–mediated inflammation by cleaving complement components C3 and C5, enhancing Toll-like receptor 4 (TLR4) signaling, and activating PARs, thereby promoting cytokine release and altering the inflammatory microenvironment [31,32,33,34,35,36,37,38]. uPAR expression is upregulated upon T cell activation and integrin engagement through signaling pathways that involve both protein kinase C (PKC) activation and increased intracellular cyclic AMP (cAMP) [39,40]. Surface uPAR enhances T cell migration and invasion—particularly through fibrin and Matrigel matrices—and facilitates tissue infiltration via its interaction with uPA [41]. While several studies have investigated plasmin and uPAR in T-cell–mediated responses, direct evidence for the roles of uPA and α2AP in T cells is currently lacking. uPA deficiency has been associated with altered immune responses in mouse models, but whether this reflects a T-cell–intrinsic mechanism remains unclear. Similarly, α2AP is the principal inhibitor of plasmin in plasma, yet no study to date has demonstrated a direct regulatory role of α2AP in T cells. These gaps underscore the need for future investigations to clarify whether uPA and α2AP exert antagonistic effects in T-cell biology.

### 3.2. B Cells

Plasmin supports B cell function by facilitating hematopoietic stem cell mobilization and modulating antibody production. In conditions such as B-cell acute lymphoblastic leukemia (B-ALL), annexin A2–mediated plasmin activation promotes ECM degradation and disease progression, effects that can be mitigated by plasmin inhibition [42,43,44]. Additionally, PAP complex enhances IgG and IgM secretion, while α2AP blockade reduces autoantibody production in SSc model mice [45,46].

### 3.3. Innate Immune Cells

Fibrinolytic factors also regulate the recruitment and function of innate immune cells. Plasmin promotes monocyte chemotaxis, macrophage phagocytosis, neutrophil activation, and the secretion of cytokines and growth factors [32,47,48,49,50]. The uPA/uPAR axis enhances these processes through signaling via PAR-1 and annexin A2 [4,36,51,52], while uPAR’s interactions with integrins and LRP modulate cell adhesion and migration [13]. Moreover, uPAR influences innate immunity by positively modulating TLR signaling, particularly TLR2 and TLR4 (and possibly TLR7), thereby enhancing NF-κB activation and pro-inflammatory cytokine expression in macrophages, neutrophils, and other immune cells [53,54,55,56]. Additionally, suPAR, which is released during inflammation, serves as a biomarker of immune activation [18]. In inflamed tissues, activated neutrophils directly promote fibrin deposition by forming neutrophil extracellular traps (NETs) that scaffold platelets and activate the contact pathway to accelerate thrombin generation; by releasing elastase and cathepsin G that proteolytically inactivate tissue factor pathway inhibitor; and by producing myeloperoxidase (MPO)–derived oxidants that modify fibrin (ogen) and yield lysis-resistant clots [57]. Monocytes/macrophages likewise foster fibrin accumulation by up-regulating tissue factor (TF) and shedding TF-bearing microvesicles that amplify local thrombin generation, by supplying factor XIII-A (FXIII-A) that cross-links and stabilizes fibrin, and by producing PAI-1, thereby creating an antifibrinolytic milieu [58,59]. Beyond its antifibrinolytic function, α2AP contributes to immune regulation by promoting the production of interleukin-1β (IL-1β) and tumor necrosis factor-α (TNF-α), as well as by regulating neutrophil and lymphocyte recruitment and IgE expression, highlighting its multifaceted immunomodulatory role [60,61,62,63,64]. Similarly, PAI-1 enhances the production of pro-inflammatory cytokines such as TNF-α and IL-6, and promotes immune cell infiltration and metabolic regulation during tissue injury [28].

These immunomodulatory effects suggest that dysregulation of the fibrinolytic system—either through hyperactivation or inhibition—may contribute to pathological immune responses, including chronic inflammation, or autoimmunity. Targeting specific fibrinolytic factors may offer novel strategies to modulate immune responses in autoimmune diseases, chronic inflammatory conditions, and tissue injury.

### 3.4. Cytokine Regulation of Fibrinolysis

Inflammatory cytokines differentially regulate individual components of the fibrinolytic system. Plg is synthesized predominantly in the liver, and its expression is induced by interleukin-6 (IL-6) [65,66]. The urokinase axis is generally up-regulated by pro-inflammatory cytokines: TNF-α and IL-1β increase uPA and uPAR expression across multiple cell types, including monocytes, neutrophils, epithelial cells, and endothelial cells [67,68,69]. In contrast, endothelial tPA is consistently down-regulated by TNF-α, thereby limiting plasmin generation [70]. Among the physiological inhibitors, PAI-1 is robustly induced by TNF-α, IL-1β, IL-6, and TGF-β in diverse cell types such as endothelial cells, fibroblasts, and macrophages [28,71]. Evidence on α2AP is more limited; however, our group has shown that interferon-γ (IFN-γ) increases α2AP production, thereby reinforcing antifibrinolysis [60]. Taken together, these data indicate that TNF-α, IL-1β, IL-6, and TGF-β tend to suppress fibrinolysis through convergent PAI-1 induction, TNF-α–mediated down-regulation of endothelial tPA, and increased α2AP, whereas IFN-γ can exert context-dependent effects but strengthens antifibrinolysis via α2AP.

## 4. Systemic Autoimmune Diseases and the Fibrinolytic System

### 4.1. Rheumatoid Arthritis

Rheumatoid arthritis (RA) is a chronic, systemic autoimmune disease characterized by recurrent inflammation primarily affecting the synovium, as well as the cartilage, bone, and surrounding musculature. A hallmark clinical feature of RA is morning stiffness, which is often accompanied by joint pain and functional impairment. These symptoms are most pronounced in the early morning and are essential for the clinical diagnosis of RA [72]. Advances in antirheumatic therapies, particularly biologic agents such as tocilizumab, have demonstrated efficacy not only in reducing inflammation but also in ameliorating haemostatic imbalances associated with RA [73].

RA is associated with a hypercoagulable state, as evidenced by elevated levels of PAP, D-dimer, fibrinogen, and FDPs [74,75,76,77,78]. These abnormalities reflect disturbances in the coagulation–fibrinolysis system, which contribute to disease progression and joint pathology. Histological analyses reveal significant infiltration of polymorphonuclear neutrophils (PMNs) and macrophages into the synovial tissue. These immune cells contribute to the formation of fibrin deposits enmeshed with neutrophils, which are resistant to fibrinolysis and help sustain persistent synovial inflammation. Notably, this impaired fibrinolysis is closely linked to morning stiffness, a hallmark symptom of RA [79].

Several fibrinolytic factors are dysregulated in RA (Table 1). Studies have demonstrated significantly increased expression of uPA, uPAR, and PAI-1, along with decreased expression of tPA in the synovial tissue, synovial fluid, and plasma of RA patients [80,81,82,83,84].

Insights from animal models further elucidate the roles of fibrinolytic factors in RA pathogenesis. Mice lacking uPA exhibit excessive fibrin accumulation and more severe arthritis, while PAI-1-deficient mice demonstrate reduced fibrin deposition and milder disease manifestations [80,85,86,87]. Similarly, the absence of tPA exacerbates joint pathology in mice, indicating that tPA is a key mediator of fibrinolysis in RA [88].

**Table 1:** Altered Expression and Roles of Fibrinolytic Factors in RA.

Fibrinolytic Factor	Expression Change in RA	Role in RA Pathogenesis	Evidence/Notes	Refs
uPA	Increased expression in synovial tissue, synovial fluid and plasma	Promotes fibrinolysis and modulates immune responses; Deficiency leads to excessive fibrin and worsened arthritis	uPA knockout mice show severe arthritis	[80,81,82,83,86]
uPAR	Increased expression in synovial tissue	Localizes uPA activity at the cell surface; Involved in pericellular proteolysis and immune cell migration	Supports uPA function and inflammatory responses	[80,84]
PAI-1	Increased expression in synovial tissue	Inhibits uPA and tPA; Deficiency reduces fibrin deposition and arthritis severity	PAI-1 knockout mice show milder disease	[80,82,87]
tPA	Increased expression in synovial fluid and plasma	Promotes fibrin degradation; Deficiency aggravates joint inflammation	tPA knockout mice show exacerbated pathology	[80,81,82,86,88]
Plg	Not about expression, but function required	Essential for fibrin degradation and RA onset; Deficiency prevents arthritis onset post-immunization	Plg knockout mice do not develop arthritis	[89,90]

Plg is essential for the development of RA symptoms. In Plg-deficient mice, arthritis does not develop following collagen immunization, suggesting that Plg activity is required for disease onset [89,90]. However, the role of Plg appears to be joint-specific; Plg deficiency leads to increased fibrin accumulation in both the paws and knees, but with different pathological outcomes.

Collectively, these findings indicate that RA progression is driven by an imbalance in fibrinolytic activity, leading to impaired fibrin clearance, sustained inflammation, and functional deficits (Table 1). Modulating fibrinolytic pathways may therefore offer novel therapeutic opportunities to restore immune and tissue homeostasis in RA.

### 4.2. Systemic Lupus Erythematosus

Systemic lupus erythematosus (SLE) is a chronic autoimmune disease characterized by widespread inflammation and immune-mediated damage to multiple organ systems, including the skin, joints, kidneys, cardiovascular system, and central nervous system [91]. Common clinical manifestations include fatigue, fever, arthralgia, malar rash, renal involvement (e.g., lupus nephritis (LN)), and neurological symptoms such as headaches, cognitive dysfunction, and seizures [92]. SLE follows a relapsing–remitting course, and although no definitive cure exists, immunosuppressive therapies, including corticosteroids, are used to manage disease activity and improve patient outcomes.

SLE is associated with dysregulation of the fibrinolytic system, contributing to a prothrombotic state [8,93]. Damage to endothelial cells, a hallmark of SLE, impairs fibrinolytic capacity and promotes hypercoagulability. Systemic inflammation during active disease phases increases fibrinogen levels, leading to the formation of denser and more highly cross-linked fibrin networks that are resistant to enzymatic degradation [35]. Elevated D-dimer levels, observed even in patients with low to moderate disease activity, reflect increased fibrin turnover and are significantly higher than in healthy individuals [94,95]. SLE patients demonstrate increased plasma concentrations of PAP and α2AP, indicating ineffective fibrinolytic activity [96,97]. Additionally, antiphospholipid antibodies (APAs), commonly present in SLE, further impair normal fibrinolysis and substantially elevate the risk of venous and arterial thrombosis [98].

Dysregulation of specific fibrinolytic factors has also been observed (Table 2). Levels of tPA are often decreased, while von Willebrand factor (vWF) antigen levels are elevated, indicating endothelial dysfunction [99]. These changes favor the formation of rigid, less degradable fibrin clots, increasing susceptibility to thrombotic complications [100]. Preclinical studies using lupus-prone mouse models support a protective role for Plg in modulating disease severity. Deficiency of Plg exacerbates autoantibody production, renal inflammation, and glomerular injury, while reduced plasmin activity promotes the formation of lysis-resistant fibrin deposits [101,102]. Enhancing Plg activation may attenuate systemic inflammation and reduce thrombotic risk, representing a promising therapeutic strategy in SLE management [103,104]. Elevated levels of α2AP and PAP have also been observed in lupus models. Notably, α2AP deficiency has been shown to mitigate pristane-induced renal pathology, including mesangial expansion, collagen deposition, fibrin accumulation, and pro-inflammatory cytokine expression in mice, highlighting α2AP as a potential therapeutic target [39]. suPAR has emerged as a valuable biomarker in SLE, particularly in cases involving LN. Plasma and urinary suPAR levels are significantly elevated in patients with active LN compared to those in remission or healthy controls [105,106,107]. In vitro studies further show that glomerular endothelial cells exposed to serum from patients with newly diagnosed SLE exhibit increased expression of PAI-1 and tPA, with levels correlating positively with disease activity and inversely with complement C4 levels [108].

Taken together, these findings indicate that dysregulation of the fibrinolytic system is a key contributor to both vascular and inflammatory pathologies in SLE (Table 2). Modulating specific fibrinolytic components—such as enhancing Plg activation or inhibiting α2AP—may offer novel therapeutic strategies to mitigate disease severity and reduce thrombotic risk in SLE.

### 4.3. Systemic Sclerosis

Systemic sclerosis (SSc), also known as scleroderma, is a chronic autoimmune disease characterized by excessive collagen deposition and fibrosis of the skin and internal organs [109]. The pathogenesis of SSc involves three core mechanisms: vascular injury, immune dysregulation, and excessive ECM accumulation. Vascular damage often presents early with Raynaud’s phenomenon, while immune dysfunction includes the generation of autoantibodies against nuclear antigens. These processes culminate in progressive fibrosis affecting the skin, lungs, heart, kidneys, and gastrointestinal tract.

SSc is clinically classified into two main subtypes: limited cutaneous SSc (lcSSc), in which skin involvement is restricted to the face and distal extremities, and diffuse cutaneous SSc (dcSSc), which involves more widespread skin thickening, including the trunk and proximal limbs. Common symptoms include skin thickening, joint pain, and stiffness. Internal organ fibrosis can lead to serious complications such as interstitial lung disease (ILD), pulmonary arterial hypertension (PAH), scleroderma renal crisis, and gastrointestinal dysmotility. While no curative treatment exists, immunosuppressive agents, vasodilators, and physical therapy can alleviate symptoms and improve quality of life.

SSc is associated with profound endothelial dysfunction, impaired angiogenesis, and abnormalities in coagulation and fibrinolysis [6]. A key feature is a prothrombotic state resulting from both excess fibrin deposition and deficient fibrinolysis. Plasma levels of D-dimer and PAP are significantly elevated in SSc patients, particularly in those with macrovascular complications such as peripheral ischemia [110,111].

α2AP, the principal physiological inhibitor of plasmin, is notably upregulated in SSc dermal fibroblasts, contributing to fibrosis and vascular pathology [46,112,113]. In vitro and animal studies have shown that α2AP blockade attenuates collagen deposition, immune activation, and vascular injury, highlighting its potential as a therapeutic target. The regulation of α2AP involves MMP-3, which proteolytically inactivates α2AP [113,114]. However, in SSc patients, MMP-3 activity is suppressed due to elevated levels of anti-MMP-3 autoantibodies and increased expression of tissue inhibitor of metalloproteinase-1 (TIMP-1), thereby reducing α2AP degradation [115,116]. Furthermore, neutralizing α2AP in mouse models of SSc reduces the production of anti-Scl-70 antibodies, suggesting an immunomodulatory role for α2AP [46].

uPAR deficiency leads to perivascular, dermal, and pulmonary fibrosis in mice [117,118], supporting the importance of uPAR in the progression of fibrosis. Elevated levels of suPAR have been observed in patients with SSc and are correlated with disease severity [119]. Notably, patients with dcSSc exhibit higher suPAR levels compared to those with lcSSc, and these levels are positively associated with anti-Scl-70 antibody positivity. Additionally, suPAR concentrations correlate with pulmonary function abnormalities indicative of fibrosis, microvascular complications, including Raynaud’s phenomenon, digital ulcers, nailfold capillary abnormalities, and articular involvement.

Collectively, these findings indicate that fibrinolytic imbalance in SSc contributes not only to fibrosis and vascular pathology but also to immune modulation (Table 3). Therapeutic strategies aimed at restoring fibrinolytic equilibrium—such as α2AP inhibition or modulation of uPAR signaling—may offer promising approaches to limit disease progression and target the underlying fibrotic and immunological mechanisms of SSc.

### 4.4. Sjögren’s Syndrome

Sjögren’s syndrome (SS) is a chronic autoimmune disease that primarily affects the body’s moisture-producing glands, leading to dryness in various parts of the body. The pathology of SS involves several key processes: the immune system mistakenly attacks the exocrine glands that produce tears and saliva, resulting in lymphocytic infiltration, inflammation, and glandular damage. Beyond glandular involvement, SS can also affect multiple organs and systems, including the joints, skin, lungs, kidneys, and nervous system [120,121].

Common symptoms of SS include dry eyes—often described as burning, itching, or a gritty sensation—and dry mouth, which may lead to difficulty swallowing or speaking and a persistent sensation of oral dryness. Other manifestations include joint pain and swelling, dry or irritated skin, vaginal dryness, chronic dry cough, and severe fatigue. SS is classified into two main types: primary SS, which occurs in isolation, and secondary SS, which develops in association with other autoimmune diseases such as RA or SLE. Although there is currently no cure for SS, treatment strategies focus on symptom relief and improving quality of life through medications, artificial tears, saliva substitutes, and lifestyle modifications.

The fibrinolytic system plays a role in the pathogenesis of SS. In patients with primary SS, erythrocyte sedimentation rate (ESR), high-sensitivity C-reactive protein (hsCRP), and D-dimer levels were significantly higher than those in healthy controls [122]. Patients with elevated D-dimer levels also showed increased fibrinogen, immunoglobulin A, and EULAR SS Disease Activity Index (ESSDAI) scores [123]. D-dimer levels were found to be useful for distinguishing between low and moderate/high disease activity.

These findings suggest that components of the fibrinolytic system may serve not only as markers of inflammation and disease activity but also as contributors to the underlying immune and vascular pathology in SS (Table 4). Monitoring D-dimer and related factors may provide clinical insight into disease severity and aid in therapeutic decision-making.

## 5. Organ-Specific Autoimmune Diseases and the Fibrinolytic System

### 5.1. Type 1 Diabetes

Type 1 diabetes (T1D) is a chronic autoimmune disorder in which the immune system attacks and destroys insulin-producing beta cells in the pancreas, resulting in absolute insulin deficiency [124]. Insulin is essential for regulating blood glucose levels, and its absence leads to hyperglycemia. The onset of T1D is influenced by both genetic predisposition and environmental triggers, such as viral infections. Common symptoms include excessive thirst (polydipsia), frequent urination (polyuria), increased hunger (polyphagia), unexplained weight loss, fatigue, and blurred vision. If left untreated, T1D can lead to diabetic ketoacidosis (DKA), a life-threatening condition. Long-term complications may include cardiovascular disease, neuropathy, nephropathy, and retinopathy. Management requires lifelong insulin therapy, regular blood glucose monitoring, and lifestyle modifications.

T1D is also associated with alterations in the fibrinolytic system, which contribute to disease progression and complications [125]. Patients with T1D, particularly those with microvascular complications like retinopathy or nephropathy, show elevated levels of D-dimer [126]. Other coagulation and inflammation-related biomarkers—such as vWF, IL-6, TNF-α, and PAI-1—are also increased in these patients [127].

Impaired fibrinolysis is a hallmark of T1D. Elevated PAI-1 levels inhibit fibrinolytic activity, promoting a pro-thrombotic state and increasing the risk of vascular complications. Other fibrinolytic impairments include increased incorporation of α2AP into fibrin clots [128], elevated FDPs [129], and higher circulating levels of α2AP, particularly in those with diabetic nephropathy (DN) [130]. Children and adolescents with T1D also exhibit significantly elevated PAI-1 levels compared to healthy peers [131]. In STZ-induced diabetic mouse models, α2AP deficiency, PAI-1 deficiency, and treatment with PAI-1 inhibitors have demonstrated renoprotective effects [132,133,134]. In Plg-deficient diabetic mice, albuminuria is worsened, although baseline blood pressure remains unchanged. Interestingly, angiotensin II (ANGII) increases blood pressure in wild-type and diabetic control mice but not in diabetic Plg deficient mice, suggesting a complex interaction between the fibrinolytic system and blood pressure regulation in T1D [135]. Additionally, uPAR has emerged as an important factor in T1D complications. Elevated suPAR levels have been linked to the development of chronic kidney disease (CKD) and microalbuminuria, an early marker of DN [136,137,138]. Targeting this pathway with agents like UPARANT, which inhibits the binding of uPAR to formyl peptide receptors, has shown therapeutic potential in diabetic kidney injury models [139].

Collectively, these findings indicate that dysregulation of the fibrinolytic system in T1D contributes to both systemic inflammation and organ-specific complications (Table 5). Modulating fibrinolytic activity—particularly through inhibition of PAI-1, α2AP, or uPAR signaling—holds promise as a novel therapeutic approach to reduce the vascular burden associated with T1D.

### 5.2. Inflammatory Bowel Disease (Ulcerative Colitis and Crohn’s Disease)

Inflammatory bowel disease (IBD) is a group of chronic inflammatory conditions primarily affecting the gastrointestinal (GI) tract, with ulcerative colitis (UC) and Crohn’s disease (CD) being the two main subtypes [140,141,142]. Both conditions involve an inappropriate immune response against the GI tract, influenced by genetic and environmental factors such as infections. While UC is limited to the colon and rectum and affects only the innermost mucosal layer, CD can involve any part of the GI tract from mouth to anus and features transmural (full-thickness) inflammation. These differences lead to distinct clinical manifestations and complications.

Symptoms shared by UC and CD include diarrhea, abdominal pain, fatigue, and weight loss. However, bloody diarrhea, rectal bleeding, and urgency to defecate are more common in UC, whereas fever and malabsorption-related symptoms are more typical in CD. If not properly managed, complications can arise: UC may lead to toxic megacolon and colonic perforation, while CD is more often associated with intestinal strictures, fistulas, and abscesses. Both diseases carry an increased long-term risk of colorectal cancer.

Management of IBD focuses on reducing inflammation, alleviating symptoms, and maintaining long-term remission. Treatment options include anti-inflammatory drugs, immunosuppressants, and biologic therapies. In severe or treatment-resistant cases, surgical interventions—such as colectomy in UC or resection of affected bowel segments in CD—may be required. Additionally, dietary modifications and stress management can help support symptom control and improve quality of life.

The fibrinolytic system, responsible for the degradation of fibrin clots, plays a key role in IBD pathophysiology. In both UC and CD, chronic intestinal inflammation activates both coagulation and fibrinolysis, resulting in a hypercoagulable state and increased risk of thromboembolic events such as deep vein thrombosis and pulmonary embolism [143,144,145,146].

Elevated levels of D-dimer are found in patients with active CD and UC, and they are correlated with disease activity [145,147]. Notably, D-dimer and PAP levels remain elevated even in clinically inactive IBD, suggesting a persistent imbalance in coagulation and fibrinolysis [147]. Additional hemostatic markers such as fibrinogen, prothrombin time (PT), factor V, factor VIII, and thrombocyte levels are often increased in IBD patients [148]. Interestingly, specific coagulation abnormalities differ between UC and CD. For instance, factor V, factor VIII, and PT levels are elevated exclusively in UC; Plg levels are significantly increased only in CD, especially during active disease; and thrombocyte and fibrinogen levels are elevated in both UC and CD [148].

Moreover, pro-inflammatory cytokines such as TNF-α and IL-6—frequently elevated in CD—can further influence the fibrinolytic system [146]. High levels of white blood cells (WBC) and hsCRP also correlate with active disease and fibrinolytic activity [144]. Genetic predisposition may play a role in IBD-associated hypercoagulability. The factor V Leiden mutation, a known thrombophilic risk factor, was found in 1.96% of UC patients, 16.66% of CD patients, and 3.84% of healthy controls [148].

Several key regulators of fibrinolysis are altered in IBD (Table 6): PAI-1 levels are consistently elevated in the serum, mucosa, and feces of UC and CD patients, contributing to impaired fibrinolysis [149,150]. Conversely, tPA levels are significantly decreased in IBD patients compared to healthy controls [151]. uPA is markedly elevated in inflamed UC tissues and correlates with disease severity. uPA is expressed in neutrophils within inflamed colorectal tissue [152].

Evidence from animal models supports the pathogenic role of uPA in IBD. In uPA deficient mice, colitis severity was significantly reduced compared to wild-type mice. Similarly, the administration of UK122, a selective uPA inhibitor, in DSS-induced colitis models results in lower disease activity index and improved histological scores, suggesting that targeting the uPA system may offer therapeutic benefits [152]. In IBD, hypoxia-inducible factor (HIF)-dependent nuclear factor of activated T cells 1 (NFATC1) activation promotes uPAR expression in the intestinal epithelium [153]. Both uPA and uPAR are significantly upregulated during epithelial barrier breakdown and downregulated during barrier repair. Blocking their interaction, either genetically or pharmacologically, helps preserve barrier integrity. uPAR deficiency enhances EGF/EGFR signaling, supports epithelial recovery, and improves barrier function both in vitro and in colitis-induced mice [154]. Furthermore, in mice, uPAR expression increases as colitis progresses. Interestingly, uPAR deficient mice exhibit more severe inflammation than wild-type mice, as shown by clinical assessment, endoscopic imaging, and colon histology.

Collectively, these findings highlight the complex and context-dependent roles of fibrinolytic factors in IBD pathogenesis—modulating coagulation, inflammation, immune responses, and tissue repair (Table 6). Therapeutic strategies targeting fibrinolytic pathways, particularly PAI-1, uPA, and uPAR, may offer novel strategies to control disease activity and reduce complications in IBD.

### 5.3. Graves’ Disease

Graves’ disease (GD) is an autoimmune disorder and is the most common cause of hyperthyroidism. It is characterized by the production of thyroid-stimulating immunoglobulins (TSIs), which bind to and activate thyroid-stimulating hormone (TSH) receptors on thyroid follicular cells. This activation leads to excessive secretion of thyroid hormones, resulting in symptoms such as an increased metabolic rate, tachycardia, weight loss, nervousness, irritability, hand tremors, heat intolerance, excessive sweating, and fatigue. Other clinical features include goiter, menstrual irregularities, reduced libido or erectile dysfunction, frequent bowel movements, and sleep disturbances [155,156]. In addition to systemic symptoms, GD can cause Graves’ ophthalmopathy, an inflammatory condition involving periorbital tissues that leads to bulging eyes. In rare cases, Graves’ dermopathy may occur, characterized by localized skin thickening and discoloration, particularly over the shins or feet.

Although there is no definitive cure, treatments such as anti-thyroid drugs, radioactive iodine therapy, and thyroidectomy aim to reduce thyroid hormone production and control symptoms. Supportive therapies, including β-blockers and lifestyle modifications, also play a role in symptom management.

GD is frequently associated with a hypercoagulable state, reflecting an increased risk of thromboembolic events. Elevated thyroid hormone levels can influence the synthesis and activity of various coagulation factors and impair the function of endothelial cells [155,157]. This dysfunction disrupts the normal balance between coagulation and fibrinolysis, reducing the body’s ability to dissolve clots and thereby promoting thrombosis [155,157]. Patients with GD often exhibit reduced fibrinolytic activity, as evidenced by elevated levels of PAI-1 and other prothrombotic markers. Notably, increased PAI-1 levels have been detected in the tears of patients with Graves’ orbitopathy, suggesting localized inhibition of fibrinolysis in orbital tissues [158,159]. Increased levels of α2AP have also been observed in patients with GD, and there is evidence suggesting a link between α2AP and thromboembolic complications in GD [160]. Additionally, α2AP has been associated with cerebrovascular thromboembolism [161], highlighting its role in the imbalance between coagulation and fibrinolysis.

Overall, these findings suggest that dysregulation of the fibrinolytic system plays a significant role in the pathogenesis and complications of GD (Table 7). Monitoring coagulation profiles in addition to thyroid function may be important for comprehensive patient management. Furthermore, components of the fibrinolytic pathway—particularly PAI-1 and α2AP—may serve as potential biomarkers or therapeutic targets in GD.

## 6. Secondary Diseases Related to Autoimmune Diseases and the Fibrinolytic System

### 6.1. Macrophage Activation Syndrome

Macrophage activation syndrome (MAS) is a severe, potentially life-threatening hyperinflammatory disorder characterized by the uncontrolled activation of macrophages and T lymphocytes. This excessive immune activation results in a cytokine storm and subsequent multi-organ dysfunction. Common clinical features include high fever, hepatosplenomegaly, cytopenias, and markedly elevated ferritin levels. MAS frequently occurs in association with autoimmune diseases such as systemic juvenile idiopathic arthritis (sJIA) and SLE [162]. Diagnosis is based on clinical presentation and laboratory findings, often supported by bone marrow examination showing hemophagocytosis [162]. Prompt and aggressive immunosuppressive treatment—typically corticosteroids, cyclosporine, and biologics targeting specific cytokines—is essential due to the syndrome’s rapid progression and high mortality [163].

The fibrinolytic system plays a critical role in MAS pathophysiology through its interaction with inflammatory and coagulation pathways. MAS disrupts fibrinolytic homeostasis, resulting in either a hypercoagulable state or a tendency toward bleeding. Elevated levels of pro-inflammatory cytokines, including interferon-γ (IFN-γ), TNF-α, and IL-6, contribute to this dysregulation [164,165,166]. Fibrinolysis markers such as D-dimer and PAP are significantly elevated, reflecting excessive fibrinolysis [164,165,166].

Experimental models of MAS have demonstrated that α2AP plays a pathological role in promoting macrophage accumulation, liver injury, and fibrin deposition. Notably, α2AP deficiency attenuates these pathological features in mouse models of MAS [167]. Additionally, excessive plasmin activity has been observed during MAS progression. Plasmin not only contributes to tissue destruction and hemorrhagic complications but also amplifies inflammatory responses by promoting the recruitment of inflammatory cells and enhancing cytokine and chemokine production [4]. Genetic or pharmacological inhibition of plasmin effectively mitigated MAS-associated lethality and reduced cytokine peaks in animal models, indicating a critical role for plasmin in driving disease severity [168].

These findings highlight that MAS is characterized by complex fibrinolytic dysregulation, where both α2AP and plasmin play important but different roles in driving disease severity. Therapeutically targeting these factors may help control inflammation, reduce coagulation-related complications, and improve patient outcomes.

### 6.2. Antiphospholipid Syndrome

Antiphospholipid syndrome (APS) is defined by the presence of antiphospholipid antibodies (aPL) in patients with thrombotic complications and/or adverse pregnancy outcomes [169,170]. It is a major cause of acquired thrombophilia, affecting both arterial and venous circulation, particularly in young individuals [171]. Thrombotic events are the most frequent manifestations and the leading cause of death in APS [172].

APS can develop on its own (primary APS) or together with other autoimmune diseases, most commonly SLE [173]. Thrombotic APS is characterized by venous, arterial, or microvascular thrombosis, while obstetric APS presents with pregnancy-related complications [174].

Despite advances in understanding its pathogenesis, the mechanisms underlying APS remain incompletely elucidated. Treatment still relies primarily on anticoagulants, which are more effective against venous thrombosis than microvascular complications [174]. APS remains associated with significant morbidity and mortality [172], underscoring the need for improved therapeutic strategies.

Key aPLs include anticardiolipin (aCL), lupus anticoagulant (LA), and anti-β2-glycoprotein I (anti-β2GPI), with β2GPI being the principal antigenic target [169,170]. These antibodies are central to the pathogenesis of thrombosis in APS [169,174]. β2GPI, also known as apolipoprotein H, plays multiple roles in both coagulation and fibrinolysis. It may promote coagulation by binding to thrombin and protecting it from inactivation by heparin cofactor II, as well as by inhibiting the inactivation of activated factor V by activated protein C (APC) [175]. In the context of fibrinolysis, β2GPI exhibits both pro- and antifibrinolytic properties. It can act as a cofactor for tPA-mediated plasmin generation, but it also forms a complex with Plg that inhibits plasmin formation, thereby establishing a negative feedback mechanism within the fibrinolytic system [176,177,178].

Annexin A2, a receptor for β2GPI on endothelial cells, is essential for cell-surface fibrinolysis through its ability to bind both tPA and Plg [179,180,181]. In APS, both anti-β2GPI and anti-annexin A2 antibodies have been reported. These antibodies impair A2-mediated plasmin generation, reduce endothelial fibrinolysis, and promote thrombosis in vivo [182].

These findings highlight the interplay between coagulation, fibrinolysis, and autoantibody-mediated endothelial dysfunction in APS. Targeting these mechanisms may offer therapeutic strategies beyond anticoagulation, particularly for managing microvascular complications.

## 7. Integrative Discussion and Therapeutic Perspective

Across autoimmune diseases, several recurrent expression patterns can be identified (Table 8). PAI-1 and α2-antiplasmin are frequently upregulated in systemic and organ-specific conditions—including RA, SLE, SSc, T1D, and GD—consistent with hypofibrinolysis and fibrin persistence. By contrast, uPA and uPAR tend to be elevated within inflamed tissues (e.g., synovium or intestinal mucosa) across RA, MS, and IBD, facilitating leukocyte trafficking and tissue infiltration. Decreases in circulating Plg have been reported in SLE and APS and associated with thrombotic risk. Plasmin activity generally tracks with this hypofibrinolytic profile, whereas tPA shows disease- and compartment-specific variability. Taken together, these profiles suggest that some fibrinolytic components (e.g., PAI-1, α2-antiplasmin) act as broad indicators of impaired fibrinolysis, whereas others (e.g., uPA/uPAR) more closely reflect disease-specific immune activation and tissue invasion. These trends highlight candidate biomarkers for disease monitoring and nominate pathway nodes for therapeutic targeting.

Suppression of the fibrinolytic system—as observed in SLE, SSc, T1D, and GD—is characterized by elevated levels of PAI-1 and α2AP. This leads to decreased plasmin activity, accumulation of fibrin, and impaired ECM degradation, which may delay the resolution of inflammation and promote chronic inflammation and defective tissue repair. In contrast, hyperactivation of the fibrinolytic system—as seen in RA and IBD—is associated with increased expression of tPA and uPA, along with persistently high levels of PAI-1 and α2AP. This coexistence of activation and inhibition may result in excessive ECM degradation and enhanced activation of cytokines and MMPs, thereby promoting immune cell infiltration and tissue destruction.

**Figure 2:**
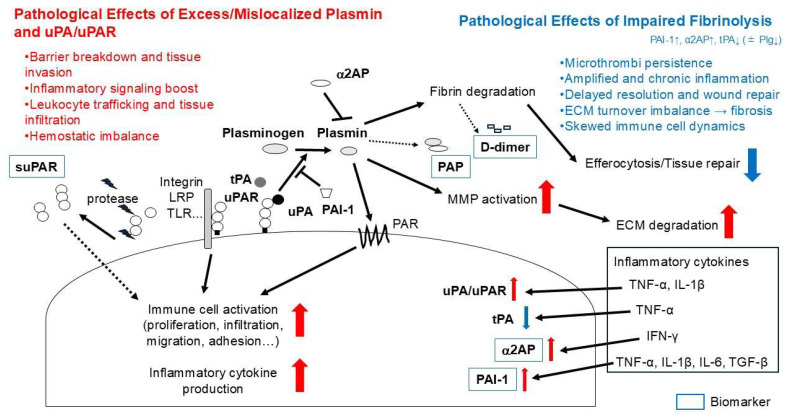
Pathogenic Outcomes of Fibrinolytic Imbalance. Excess or mislocalized plasmin and uPA/uPAR signaling cause barrier disruption, tissue invasion, enhanced inflammation, leukocyte trafficking, and hemostatic imbalance. Impaired fibrinolysis (↑ PAI-1, ↑ α2AP, ↓ tPA, ±↓ Plg) leads to microthrombi persistence, chronic inflammation, delayed repair, ECM imbalance with fibrosis, and altered immune cell dynamics. Key molecular events include plasmin generation, inhibition by α2AP, regulation by PAI-1, and downstream effects such as MMP activation, ECM degradation, efferocytosis, and cytokine production. Circulating biomarkers include PAP, D-dimer, α2AP, PAI-1, and suPAR.

To reconcile these observations, it is useful to distinguish causality versus consequence and to consider fibrinolysis as an immune “gatekeeper.” In fibrin-rich inflammatory niches, insufficient fibrinolysis (e.g., ↑ PAI-1/α2AP, ↓ tPA) can causally stabilize fibrin scaffolds that sustain leukocyte recruitment and amplify innate signaling; conversely, appropriately timed fibrin removal is anti-inflammatory because it clears fibrin/microthrombi, facilitates efferocytosis, and promotes tissue repair. By contrast, excess or mislocalized proteolysis may secondarily exacerbate inflammation (e.g., via MMP activation or exposure of matrix epitopes) (Figure 2). Brief disease-specific dichotomy (summary): RA/IBD—timely fibrin clearance is anti-inflammatory, though exuberant proteolysis can damage tissue; SLE/SSc/T1D/GD—predominant hypofibrinolysis sustains fibrin and immune activation, so cautious enhancement of fibrinolysis is beneficial; SS—limited data suggest gland-focused hypofibrinolysis with potential benefit from partial restoration.

Together, these observations suggest that the fibrinolytic system acts as a dual regulator of immune responses. Suppression may lead to unresolved inflammation and fibrosis, while hyperactivation may amplify immune infiltration and cytokine-mediated damage. Thus, an imbalance in fibrinolytic activity—whether suppressed or overactive—may represent a core mechanism linking vascular dysfunction, immune activation, and fibrotic progression in the pathophysiology of autoimmune diseases.

From a therapeutic standpoint, targeting components of the fibrinolytic system may hold significant potential for modulating immune responses, controlling inflammation, and mitigating fibrotic changes. Modulation of key regulators—such as PAI-1, α2AP, or the uPA/uPAR axis—may help restore fibrinolytic balance and promote tissue homeostasis. However, such interventions must be tailored to disease-specific contexts, as both upregulation and suppression of the fibrinolytic pathway can be pathogenic depending on the condition.

Further research is essential to clarify the context-dependent roles of individual fibrinolytic components and to develop selective modulators capable of fine-tuning this complex system. A deeper mechanistic understanding will provide a foundation for novel therapeutic strategies targeting fibrinolytic dysfunction in autoimmune and inflammatory diseases.

## Figures and Tables

**Table 2:** Altered Expression and Roles of Fibrinolytic Factors in SLE.

Fibrinolytic Factor	Expression Change in SLE	Role in SLE Pathogenesis	Evidence/Notes	Refs
PAP	Increased in plasma	Reflects increased plasmin generation; however, fibrinolysis remains ineffective	Indicates ongoing but insufficient fibrinolytic activity	[96,97]
α2AP	Increased in plasma and lupus models	Inhibits plasmin; promotes fibrin persistence, renal inflammation, and fibrosis	α2AP deficiency reduces renal pathology (e.g., mesangial expansion, collagen/fibrin deposition)	[60,96,97]
tPA	Decreased in plasma and glomerular ECs	Impaired plasminogen activation; contributes to fibrin accumulation and thrombotic complications	Lower levels correlate with disease activity	[99,108]
PAI-1	Increased in glomerular endothelial cells	Inhibits tPA; exacerbates fibrinolytic imbalance and inflammation	Positively correlates with disease activity; inversely with complement C4	[108]
D-dimer	Elevated even with low/moderate activity	Reflects enhanced fibrin turnover and thrombotic risk	Higher than in healthy individuals	[94,95]
Plg	Functional activity reduced in models	Essential for limiting inflammation and autoimmunity; deficiency worsens glomerular injury and autoantibody production	Enhancing Plg activation reduces systemic inflammation and thrombosis	[101,102,103,104]
suPAR	Elevated in plasma and urine	Biomarker for lupus nephritis; associated with active disease and glomerular injury	Elevated in active LN; correlates with disease activity	[105,106,107]

**Table 3:** Altered Expression and Roles of Fibrinolytic Factors in SSc.

Fibrinolytic Factor	Expression Change in SSc	Role in SSc Pathogenesis	Evidence/Notes	Refs
PAP	Increased in plasma	Reflects increased plasmin generation, but fibrinolysis remains ineffective; associated with macrovascular complications	Elevated in patients with peripheral ischemia	[111]
D-dimer	Increased in plasma	Indicates enhanced fibrin formation and degradation; reflects a prothrombotic state	Correlates with macrovascular involvement	[110]
α2AP	Upregulated in SSc dermal fibroblasts	Inhibits plasmin; promotes collagen deposition, fibrosis, vascular damage, and immune activation	Inhibition reduces fibrosis and restores perfusion in bleomycin-induced models; also reduces autoantibodies	[46,113]
uPAR	Deficiency leads to fibrosis (in models)	Regulates ECM remodeling and immune responses; deficiency promotes dermal and pulmonary fibrosis	Mice with uPAR deficiency develop fibrosis	[117,118]
suPAR	Elevated in plasma	Biomarker for disease severity; associated with diffuse cutaneous SSc, anti-Scl-70 positivity, pulmonary and vascular complications	Correlates with Raynaud’s, digital ulcers, pulmonary function decline, and articular symptoms	[119]

**Table 4:** Altered Expression and Roles of Fibrinolytic Factors in SS.

Fibrinolytic Factor	Expression Change in SS	Role in SS Pathogenesis	Evidence/Notes	Refs
D-dimer	Increased in primary SS patients	Indicates enhanced fibrin formation and degradation; reflects hypercoagulability and correlates with disease activity	Higher in patients with increased ESSDAI scores; useful for distinguishing moderate/high vs. low activity	[122,123]
Fibrinogen	Increased in patients with high D-dimer	Contributes to prothrombotic state and systemic inflammation	Associated with increased D-dimer and higher disease activity	[123]

**Table 5:** Altered Expression and Roles of Fibrinolytic Factors in T1D.

Fibrinolytic Factor	Expression Change in T1D	Role in T1D Pathogenesis	Evidence/Notes	Refs
D-dimer	Increased, especially with microvascular complications	Marker of fibrin turnover; reflects hypercoagulability and vascular injury	Elevated in patients with retinopathy/nephropathy	[126]
PAI-1	Increased in adults and children with T1D	Inhibits tPA/uPA, reducing fibrinolysis and promoting thrombosis	Linked to nephropathy; PAI-1 deficiency or inhibition is renoprotective in diabetic mouse models	[127,130,131,133,134]
α2AP	Increased in circulation and fibrin clots	Inhibits plasmin; promotes fibrin persistence and renal damage	Deficiency improves renal outcomes in diabetic mice; increased incorporation into fibrin clots	[128,130,132]
FDPs	Increased	Indicates increased fibrin breakdown, but impaired clearance due to inhibitory imbalance	Associated with vascular dysfunction	[129]
Plg	Deficiency worsens albuminuria in models	Essential for maintaining renal function and fibrin clearance	Diabetic Plg-deficient mice have aggravated nephropathy and loss of blood pressure response to ANGII	[135]
suPAR	Elevated, associated with CKD and albuminuria	Biomarker of early kidney disease; contributes to podocyte injury and inflammation	Therapeutic inhibition (e.g., UPARANT) ameliorates diabetic nephropathy in models	[136,137,138,139]

**Table 6:** Altered Expression and Roles of Fibrinolytic Factors in IBD.

Fibrinolytic Factor	Expression Change in IBD	Role in IBD Pathogenesis	Evidence/Notes	Refs
D-dimer	Elevated (even in inactive disease)	Marker of fibrin degradation; reflects ongoing coagulation and fibrinolysis	Correlates with disease activity; elevated in UC and CD	[145,147]
PAP	Elevated	Indicates active plasmin generation but also inhibition by α2AP	Remains high during remission; suggests persistent dysregulation	[147]
PAI-1	Elevated (serum, mucosa, feces)	Inhibits fibrinolysis, promotes thrombosis, contributes to inflammation	Strongly upregulated in both UC and CD	[149,150]
tPA	Decreased	Impaired activation of plasminogen to plasmin; reduced fibrin clearance	Lower than in healthy controls	[151]
uPA	Increased (especially in UC)	Promotes inflammation, neutrophil infiltration, epithelial barrier breakdown	Expressed in neutrophils; inhibition reduces colitis severity in mice	[152]
uPAR	Increased during inflammation	Modulates epithelial repair, immune cell signaling	Upregulated via HIF–NFATC1; KO mice show worse colitis	[153,154]
Plg	Increased (in CD only)	Source of plasmin; contributes to fibrin degradation and tissue remodeling	Elevated during active CD; less studied than uPA/uPAR	[148]

**Table 7:** Altered Expression and Roles of Fibrinolytic Factors in GD.

Fibrinolytic Factor	Expression Change in GD	Role in GD Pathogenesis	Evidence/Notes	Refs
PAI-1	Increased in plasma and orbital tissues	Inhibits fibrinolysis; contributes to prothrombotic state and localized fibrin accumulation	Elevated levels found in plasma and tears of patients with Graves’ orbitopathy	[155,157,158,159]
α2AP	Increased	Inhibits plasmin; linked to thromboembolic complications including cerebrovascular events	Associated with thrombotic risk in GD patients	[160,161]

**Table 8:** Altered expression of fibrinolytic factors and their pathogenic roles in autoimmune diseases.

Disease	Key Factors Altered	Pathogenic Roles
RA	uPA ↑, uPAR ↑, PAI-1 ↑, tPA ↑, Plg required	Hypofibrinolysis, fibrin persistence, arthritis progression
SLE	PAP ↑, α2AP ↑, tPA ↓, PAI-1 ↑, D-dimer ↑, Plg ↓, suPAR ↑	Hypofibrinolysis, renal inflammation, thrombosis, lupus nephritis
SSc	PAP ↑, D-dimer ↑, α2AP ↑, uPAR deficiency → fibrosis, suPAR ↑	Fibrosis, vascular damage, ischemia, autoantibody production
SS	D-dimer ↑, Fibrinogen ↑	Hypercoagulability, systemic inflammation
T1D	D-dimer ↑, PAI-1 ↑, α2AP ↑, FDPs ↑, Plg ↓, suPAR ↑	Thrombosis, nephropathy, vascular dysfunction, podocyte injury
IBD	D-dimer ↑, PAP ↑, PAI-1 ↑, tPA ↓, uPA ↑, uPAR ↑, Plg ↑ (CD only)	Thrombosis, impaired fibrin clearance, inflammation, barrier breakdown
GD	PAI-1 ↑, α2AP ↑	Thrombosis, fibrin accumulation, cerebrovascular events

## Abbreviations

α2AP	α2-antiplasmin
β2GPI	β2-glycoprotein I
aCL	anticardiolipin
ANGII	angiotensin II
APAs	antiphospholipid antibodies
APC	activated protein C
aPL	antiphospholipid antibodies
APS	antiphospholipid syndrome
B-ALL	B-cell acute lymphoblastic leukemia
cAMP	cyclic AMP
CD	Crohn’s disease
CKD	chronic kidney disease
dcSSc	diffuse cutaneous SSc
DKA	diabetic ketoacidosis
DN	diabetic nephropathy
ECM	extracellular matrix
EGFR	epidermal growth factor receptor
ESR	erythrocyte sedimentation rate
ESSDAI	EULAR SS disease activity index
FDPs	fibrin degradation products
FXIII-A	factor XIII-A
GD	Graves’ disease
GI	gastrointestinal
HIF	hypoxia-inducible factor
hsCRP	high-sensitivity C-reactive protein
IBD	inflammatory bowel disease
IFN-γ	interferon-γ
ILD	interstitial lung disease
lcSSc	limited cutaneous SSc
IL	interleukin
LA	lupus anticoagulant
LN	lupus nephritis
LRP	low-density lipoprotein receptor-related protein
MAS	macrophage activation syndrome
MMPs	matrix metalloproteinase
MPO	myeloperoxidase
NETs	neutrophil extracellular traps
NFATC1	nuclear factor of activated T cells 1
NF-κB	nuclear factor-κ B
NMDAR	N-methyl-D-aspartate receptor
PAH	pulmonary arterial hypertension
PAI-1	plasminogen activator inhibitor-1
PAP	plasmin–α2AP complex
PAR	protease-activated receptor
PKC	protein kinase C
Plg	plasminogen
PMNs	polymorphonuclear neutrophils
PT	prothrombin time
RA	rheumatoid arthritis
sJIA	systemic juvenile idiopathic arthritis
SLE	systemic lupus erythematosus
SS	Sjögren’s syndrome
SSc	systemic sclerosis
suPAR	soluble uPAR
T1D	type 1 diabetes
TF	tissue factor
TGF-β	transforming growth factor-β
TIMP	tissue inhibitor of metalloproteinase
TLR	toll-like receptor
TNF-α	tumor necrosis factor-α
TSH	thyroid-stimulating hormone
TSIs	thyroid-stimulating immunoglobulins
tPA	tissue-type plasminogen activator
UC	ulcerative colitis
uPA	urokinase-type plasminogen activator
uPAR	uPA receptor
VEGF	vascular endothelial growth factor
VEGFR	vascular endothelial growth factor receptor
vWF	von Willebrand factor
WBC	white blood cells
